# Influence of Adding Low Concentration of Oxygenates in Mineral Diesel Oil and Biodiesel on the Concentration of NO, NO_2_ and Particulate Matter in the Exhaust Gas of a One-Cylinder Diesel Generator

**DOI:** 10.3390/ijerph19137637

**Published:** 2022-06-22

**Authors:** Rafael R. Maes, Geert Potters, Erik Fransen, Rowan Van Schaeren, Silvia Lenaerts

**Affiliations:** 1 Antwerp Maritime Academy, Noordkasteel Oost 6, 2030 Antwerp, Belgium; geert.potters@hzs.be (G.P.); rowan.van.schaeren@hzs.be (R.V.S.); 2 Department of Bioscience Engineering, University of Antwerp, 2020 Antwerp, Belgium; silvia.lenaerts@uantwerpen.be; 3 STATUA Center for Statistics, University of Antwerp, 2610 Antwerp, Belgium; erik.fransen@uantwerpen.be

**Keywords:** oxygenates, diesel, biodiesel, NOx, particulate matter

## Abstract

Air quality currently poses a major risk to human health worldwide. Transportation is one of the principal contributors to air pollution due to the quality of exhaust gases. For example, the widely used diesel fuel is a significant source of nitrogen oxides (NOx) and particulate matter (PM). To reduce the content NOx and PM, different oxygenated compounds were mixed into a mineral diesel available at the pump, and their effect on the composition of exhaust gas emissions was measured using a one-cylinder diesel generator. In this setup, adding methanol gave the best relative results. The addition of 2000 ppm of methanol decreased the content of NO by 56%, 2000 ppm of isopropanol decreased NO_2_ by 50%, and 2000 ppm ethanol decreased PM by 63%. An interesting question is whether it is possible to reduce the impact of hazardous components in the exhaust gas even more by adding oxygenates to biodiesels. In this article, alcohol is added to biodiesel in order to establish the impact on PM and NOx concentrations in the exhaust gases. Adding methanol, ethanol, and isopropanol at concentrations of 2000 ppm and 4000 ppm did not improve NOx emissions. The best results were using pure RME for a low NO content, pure diesel for a low NO_2_ content, and for PM there were no statistically significant differences.

## 1. Introduction

Sustainability of transport is becoming a growing challenge for the future as the continued use of fossil fuel is breaking down the climatic equilibria of our planet at an increasing pace. The number of vehicles on the road, ships on water, and planes in the air continue to increase incessantly, leading to a huge rise in the quantity of exhaust gases [1,2]. However, it is maritime transport that constitutes one of the major challenges of the future. Road transport tends to or should comprise electrical vehicles on which a lot of research has already been done [3,4]. Even air transport is developing different alternative strategies for sustainable energy use [5,6]. Maritime transport is still in the process of choosing the best possible sustainable replacement, whether this is hydrogen, ammonia, LNG, or biodiesel [7,8].

This choice will hinge on two aspects of engine emissions. Firstly, the potential fuel will have to be carbon neutral, especially within the frame of climate catastrophe. Secondly, combustion engines, such as diesel engines on board ships, are bound to produce particulate matter (PM) and nitric oxides (NOx). Measurements of exhaust gas emissions of ship diesel engines have been conducted around Europe and these amounts of PM and NOx amount to about 0.2 million ton PM and 3 million ton NOx [9].

Particulate matter (PM), also known as atmospheric aerosol particles, is comprised of solid or liquid particles suspended in the atmosphere. The sources of PM can be natural, including for example volcanic eruptions or anthropogenic thus manmade, as in diesel engines, for example. The major problem with PM is the impact on human health. PM can be divided into different types: suspended, respirable, inhalable with a diameter between 2.5 and 10 microns, fine with a diameter of less than 2.5 micron, ultrafine, and soot. Due to its ability to penetrate deep into the lungs and bloodstream, PM can cause heart and lung disease and, in some cases, lead to heart attacks and/or premature death. The smallest particles with a diameter of less than 100 nanometer (nanoparticles) are even more dangerous because they can pass through cell membranes and migrate into organs. It is exactly these particles that are formed in diesel engines.

The anthropogenic sources of NOx are rather diverse. There is thermal NOx, which is formed by the reaction of atmospheric oxygen and nitrogen at high temperatures (above 1300 °C), as described by the Zeldovich mechanism [10,11]. Secondly, there is fuel NOx. This is nitrogen bound to organic compounds in the fuel that reacts with oxygen [12,13]. The reaction mechanism is quite complex and there is still considerable uncertainty about the exact reaction mechanism. Lastly, there is prompt NOx, which is formed by the reaction of atmospheric nitrogen with radicals formed during combustion. The contribution of the latter is disputed in literature [13], but most research considers the concentration of prompt NOx as very low. NOx reacts with moisture, thus forming nitric acids, and it reacts with volatile organic compounds, thus producing ozone. Both these compounds are considered to be major causes of lung disease and respiratory problems [14,15,16]. However, if the eventual goal is to find a way to lower air pollution as well as to find a new and sustainable fuel resource, biodiesel may well provide a good solution for both these aspects. The concentrations of hazardous components in the exhaust gases are lower than those of mineral diesel, and the production of biodiesel could cover the diesel fuel demand when choosing the right source of lipid production [17,18,19].

Another means of decreasing the NOx and PM concentrations in the exhaust gases is by adding oxygenates, which are molecules containing an oxygen atom in their molecular structure. The effect of adding oxygenates to mineral diesel has already been investigated quite extensively [20,21,22,23,24,25]. Biodiesel is itself already an oxygenate by way of its molecular structure. Therefore, and because biodiesel is a promising alternative for mineral diesel, it might be interesting to pose the question “What happens to the concentration of NOx and PM if an oxygenate is added to biodiesel?”. Different types of additives in biodiesel have been tested, such as, for example, diethyl ether/diesel [20] and waste-derived ethylene glycol diacetate/diesel [24]. In [23], a decrease in smoke opacity and NOx was detected and in [24] a decrease in NOx was detected. In both cases, a blend of biodiesel and diesel with the oxygenate in higher concentration was tested so B100 with an oxygenate in lower concentration as additive was not subject in this research.

A more thorough investigation of the effect of different oxygenates is therefore required. This study therefore investigates the influence of oxygenates in a more systematic way. The oxygenates were added in low concentrations to mineral diesel in order to influence the exhaust gas composition. The organic structures containing oxygen atoms are alcohols, ketones, aldehydes, ethers, and organic acids. In addition, the influence of water, which according to the definition is also an oxygenate, is measured. We only consider the molecules with the shortest possible chain length that are fluid under normal atmospheric circumstances. In each case we added 2000 ppm and 4000 ppm oxygenate to the mineral diesel and measured contents of NO, NO_2_, and PM in the exhaust gas, respectively. Next, we made a relative study by comparing the results. The aim of this study is to look at possibilities for improving the exhaust gas composition by decreasing its content of NO, NO_2_, and PM by adding low quantities of oxygenates. An additional aim is to use these results when substituting diesel for biodiesel, which already contains oxygenated compounds because biodiesel is a mixture of several (methyl)esters of fatty acids. The study focuses only on the fuel itself and does not consider motor management, such as, for example, changing the injection pressure.

The reason that CO or CO_2_ was not measured is that, as we already stated, measurements of biodiesel will also be carried out. Biodiesel is produced from vegetable oil and these oils are produced from plants, thus vegetation. If we produce biodiesel from plants grown today, the biodiesel produced from this vegetation is used only a short time later. This means that the CO_2_ produced during combustion will be used in the photosynthesis process by the vegetation as it grows. In short, we can say that the CO_2_ cycle will be closed, or at least will be closed for the larger part.

## 2. Materials and Methods

### 2.1. Engine Specification

**Figure 1:**
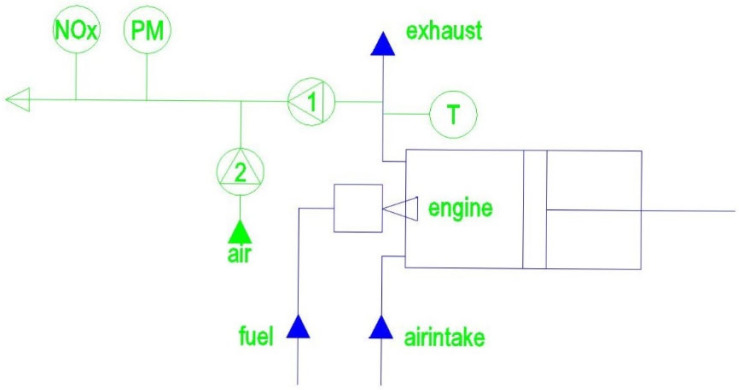
Scheme of the experimental set-up where 1 is the sampling compressor, 2 is the dilution compressor, and T is the temperature sensor. PM is the particulate matter sensor and NOx is the sensor measuring NO and NO_2_.

The experiments were conducted using a four stroke one cylinder diesel generator of type JavacNanomag NM 7500 B (KM 186FA) (Figure 1, Table 1).

In order to test the different types of fuel samples, the generator was equipped with a reservoir that could be cleaned after each measurement run. The measurements were done by way of a three-phase system. The load consisted of three identical hot air blowers by means of which the load could be increased in steps of 0.7 kW.

### 2.2. Exhaust Gas Emission

Measuring PM was done by a DustTrack DRX model 8533 (TSI instruments, Wycombe, England). The sampling time was set at 60 s and the sampling period at 10 s. The measuring range for PM is 0.001–150 mg/m^3^. Measuring occurs at a resolution of ±0.1% of reading or 0.001 mg/m^3^, whichever is greater. Measuring NO and NO_2_ was done by a Crown Con Gas-Pro (Crowncon Detection instruments Ltd., Abingdon, UK). NO and NO_2_ were measured three times during that minute. The measuring range for NO is 0–100 ppm (with a resolution of 1 ppm), and for NO_2_ it is 0.0–20.0 ppm (with a resolution of 0.5 ppm). Note that this paper will make a distinction between NO and NO_2_.

### 2.3. Measuring Protocol

The load increased in steps of 0.7 kW and at different levels of power enough time was taken for the temperature of the exhaust gas to stabilize. This means that differences of more than a few tenths of a degree were not exceeded. At level of load 0 kW (stationary), 0.7 kW, 1.4 kW, 2.1 kW, 3.5 kW, 4.9 kW, and 5.6 kW, we measured PM, NO, and NO_2_. To measure the contents of NOx and PM, a tube was created in which a constant flow of exhaust gas was taken by means of a sampling compressor. This constant flow was diluted by adding a constant flow of ambient air. To reach this goal, an air compressor at constant rotation speed was used. Measurements were taken until we reached around nominal load of the diesel generator.

### 2.4. Adding of Oxygenates

Measurements were performed with mineral diesel (B7) bought at a gas station. The same diesel was used, and 2000 or 4000 ppm oxygenate were added to the diesel. We chose from every oxygenate group the molecule with the smallest molecular structure (shortest chain length), which was in the fluid phase under measuring circumstances (ambient p around 1 bar, T around 280 K). The oxygenates chosen were alcohol, ether, ketones, aldehydes (Acros Organics, Geel, Belgium) and water, which is also an oxygenate according to the definition. An overview of some physical properties of the different oxygenates is given in Table 2.

### 2.5. Statistical Testing

**Figure 2:**
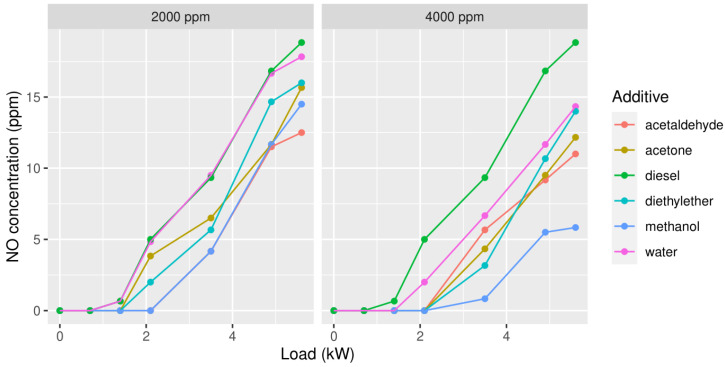
Concentrations of NO in the exhaust gas in function of the load, with 2000 ppm (**left column**) or 4000 ppm (**right column**) of each of the oxygenates added to the diesel.

**Figure 3:**
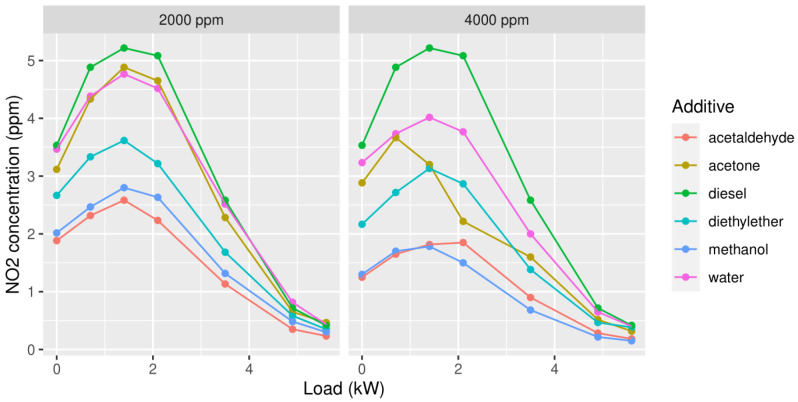
Concentrations of NO_2_ in the exhaust gas in function of the load, with 2000 ppm (**left column**) or 4000 ppm (**right column**) of each of the oxygenates added to the diesel.

**Figure 4:**
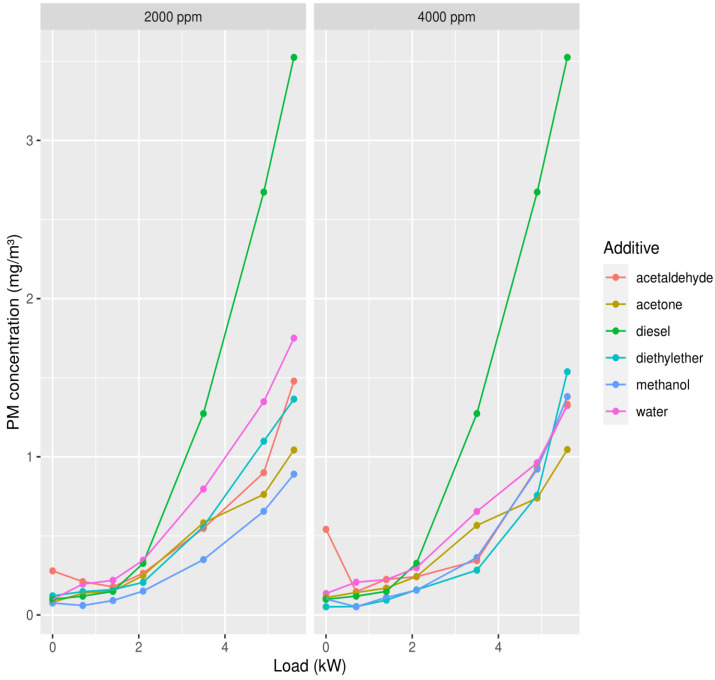
Concentrations of PM in the exhaust gas in function of the load, with 2000 ppm (**left column**) or 4000 ppm (**right column**) of each of the oxygenates added to the diesel.

**Figure 5:**
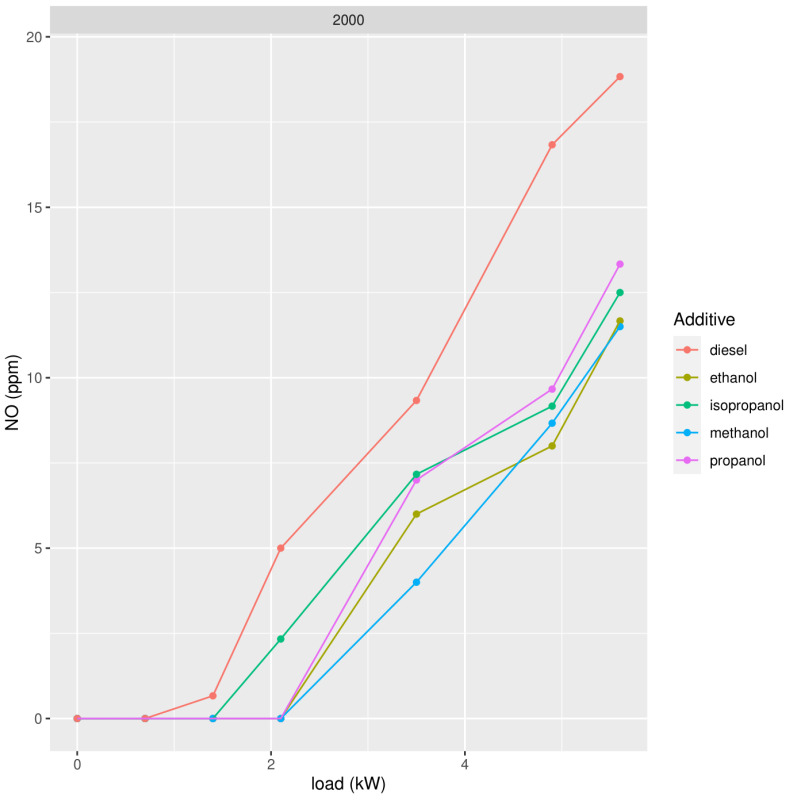
Concentration of NO in exhaust gases in function of the power of the diesel generator after addition of 2000 ppm of methanol, ethanol, propanol, and isopropanol.

**Figure 6:**
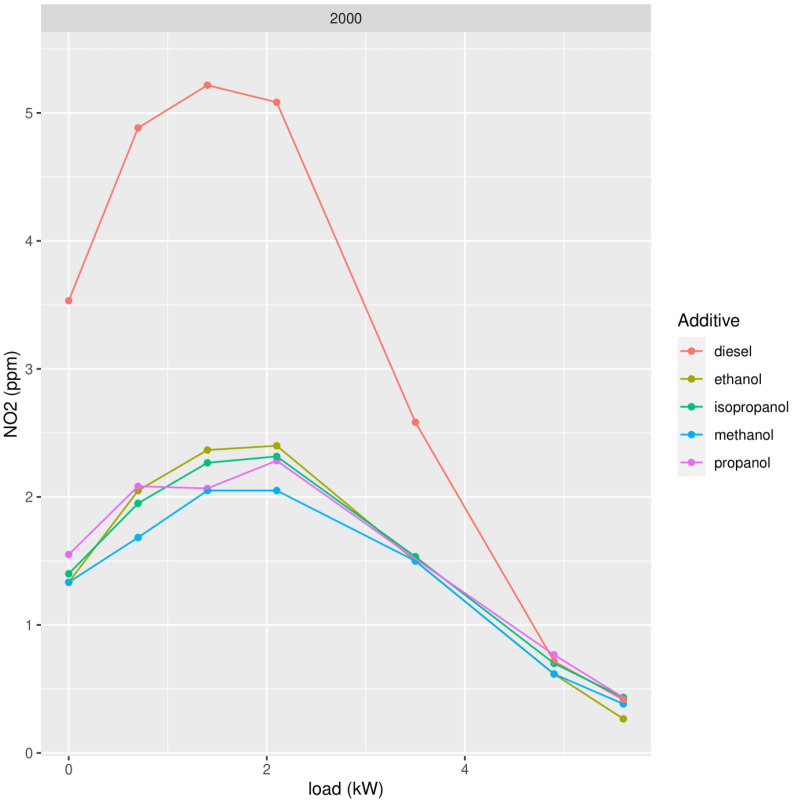
Concentration of NO_2_ in exhaust gases in function of the power of the diesel generator after addition of 2000 ppm of methanol, ethanol, propanol, and isopropanol.

**Figure 7:**
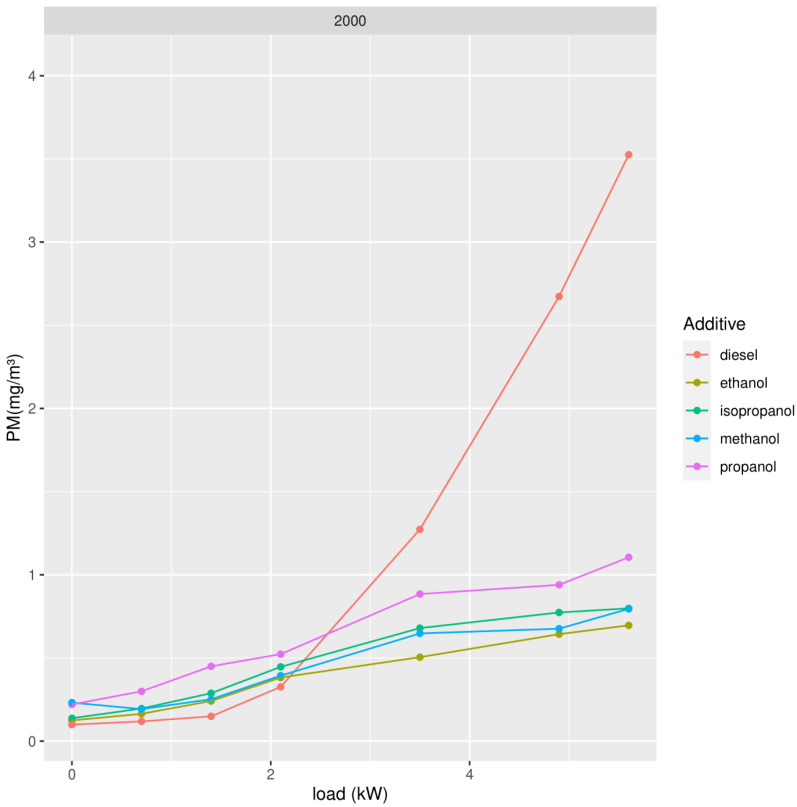
Concentration of PM in exhaust gases in function of the power of the diesel generator after addition of 2000 ppm of methanol, ethanol, propanol, and isopropanol.

**Figure 8:**
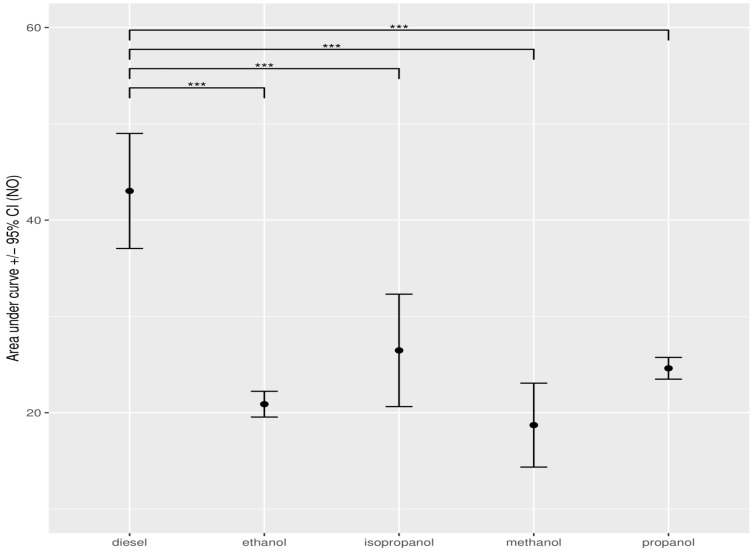
Error analysis bar chart of the concentration of NO, expressed as AUC, measured for the different alcohols between pure and diesel–alcohol mixtures. The dots show the mean AUC value, obtained through bootstrap resampling. Error bars indicate the 95% confidence interval around the mean. Asterisks show the significance of the pairwise comparison of the AUC values *** *p* < 0.001).

**Figure 9:**
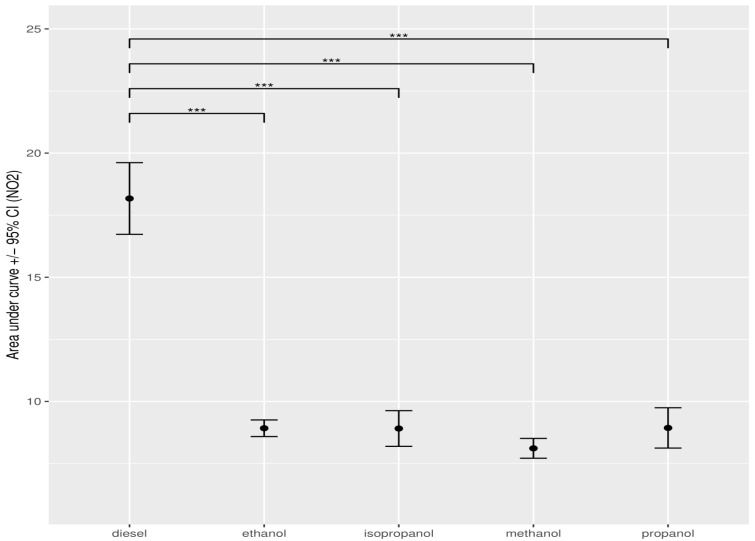
Error analysis bar chart of the concentration of NO_2_, expressed as AUC, measured for the different alcohols between pure and diesel–alcohol mixtures. The dots show the mean AUC value, obtained through bootstrap resampling. Error bars indicate the 95% confidence interval around the mean. Asterisks show the significance of the pairwise comparison of the AUC values (*** *p* < 0.001).

**Figure 10:**
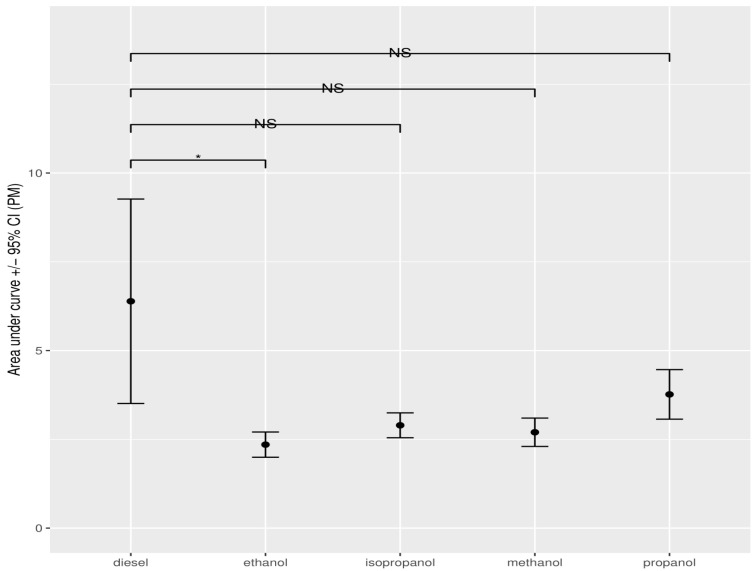
Error analysis bar chart of the concentration of PM, expressed as AUC, measured for the different alcohols between pure and diesel–alcohol mixtures. The dots show the mean AUC value, obtained through bootstrap resampling. Error bars indicate the 95% confidence interval around the mean. Asterisks show the significance of the pairwise comparison of the AUC values (* *p* < 0.05; NS: not significant).

Mean values for the area under curve (AUC) across the full range of loads were calculated for each additive, for each ppm, and for each exhaust using the trapezoid rule, as implemented in the R package DescTools. The standard deviation of the measured values allowed for the calculation of a standard deviation around the AUC using bootstrap resampling. In brief, for each exhaust 7 normal distributions (for the 7 power levels) were generated, each having the mean and standard deviation from the raw recording shown in Figures 2–10 and Figures S1–S3. Then, one random observation was picked from each of these 7 distributions and the AUC was calculated for this set of observations. This process was carried out 2000 times to obtain the mean AUC and its standard deviation.

*p*-values for pairwise comparison of exhaust between additives were generated using the test statistic of the independent sample t-test. Since only one value of the mean AUC was obtained per additive using the calculation described above, we took the standard error equal to the standard deviation. The difference in AUC value between the two additives was put in the numerator, and the denominator was the square root of the summed squared standard deviation. Under the null hypothesis of no differences between the additives, this test statistic has a t-distribution with a large number of degrees of freedom. The distribution then becomes equivalent to a standard normal distribution. For each exhaust, we calculated *p*-values for pairwise differences between either diesel or methanol, to the other additives. The *p*-value for this pairwise comparison was corrected by a Bonferroni correction for multiple hypothesis testing.

## 3. Results

### 3.1. First Measuring Campaign

#### 3.1.1. Nitrogen Oxide (NO)

The first type of emission to be tested was NO (Figure 2). At both concentrations, it can be seen that methanol gives quite good results. At 2000 ppm acetaldehyde gives slightly better results than methanol, but it should be said that the difference in temperature exceeded more than a few tenths of a degree, thus the reliability of this measurement is questionable. At 4000 ppm, we can say that the measurements were unreliable because of large differences in temperature in the exhaust. We stopped measuring with aldehyde because the engine showed knock. Although the samples were taken over the entire range, it should be noted that adding acetaldehyde is a risk even when adding small quantities. In addition, none of the additives of a significantly decreased NO exhaust compared to diesel. Statistical analysis showed that, at 4000 ppm the exhaust, when adding methanol and acetaldehyde, differs significantly from diesel.

#### 3.1.2. Nitrogen Dioxide (NO_2_)

Secondly, NO_2_ emissions were measured (Figure 3). Across both concentrations, methanol leads to a significantly decreased NO_2_. In addition, acetaldehyde at 2000 ppm shows slightly better results, but then again it should be stated that the temperature was stable, although exceeding more than a few tenths of a degree with the same conclusion as above.

#### 3.1.3. Particulate Matter (PM)

In this case, again methanol shows good results, but in the case of 4000 ppm added, ether shows slightly better results (Figure 4). Considering that in the case of NOx methanol shows a better exhaust gas composition, we should, of course, consider how alcohols influence the exhaust gas composition due to their show of overall better results.

### 3.2. Second Measuring Campaign

We repeated the measurements of the four best results, including ether and methanol at 2000 ppm and 4000 ppm obtained during the first measuring campaign in order to filter and confirm the first results for NO (Figure S1), NO_2_ (Figure S2), and PM (Figure S3). These figures can be found in supplementary data. Overall, it may be assumed that methanol 2000 ppm added gives the best results. Following a statistical analysis, a more solid conclusion will be provided.

### 3.3. Effect of Chain Length of Alcohols

We repeated the measurements using different types of the best oxygenate molecular structure, namely alcohol. We tested methanol, ethanol, propanol, and isopropanol, adding 2000 ppm to mineral diesel (Figure 5, Figure 6 and Figure 7).

In the case of alcohols, ethanol shows slightly better results than methanol to lower concentrations of PM, but for NO_x_, methanol shows slightly better results. Bearing in mind we also want to study the behavior of biodiesel which is, at this moment, still produced by transesterification with methanol (FAME: Fatty Acid Methyl Ester), the best choice for adding oxygenates to biodiesel would be methanol, provided the results when adding alcohol to biodiesel confirm this choice.

### 3.4. Statistical Data Analysis When Adding Alcohol to Diesel

To summarise the exhaust (NO, NO_2_, PM) for a given fuel mixture over the entire range of power levels, we calculated the area under curve (AUC) using the graphs in Figure 2, Figure 3, Figure 4, Figure 5, Figure 6, Figure 7 and Figures S1–S3. Standard deviations and the 95% confidence interval around the AUC were obtained by a resampling procedure, as described in the methods. Subsequently, we statistically tested whether the exhaust from the diesel with a given additive was significantly different from the exhaust from diesel alone. The results of these analyses are presented in Tables S1–S5 (Supplementary Materials). These results are visualised in Table 3 and Figure 8, Figure 9 and Figure 10.

The first measurements enable us to filter. Acetaldehyde gives rather good results, but the measurements were uncertain and too unpredictable to use. The other additives, water and acetone, did not lead to such good overall results as methanol and ether. This analysis shows that adding 2000 ppm methanol gives very good results overall. In general, the alcohol function is able to lower the concentrations of both NOx and PM when adding 2000 ppm. The next test that was performed was using other additives with an alcohol group to see if they could perform even better.

### 3.5. Addition of Oxygenates to Biodiesel

**Figure 11:**
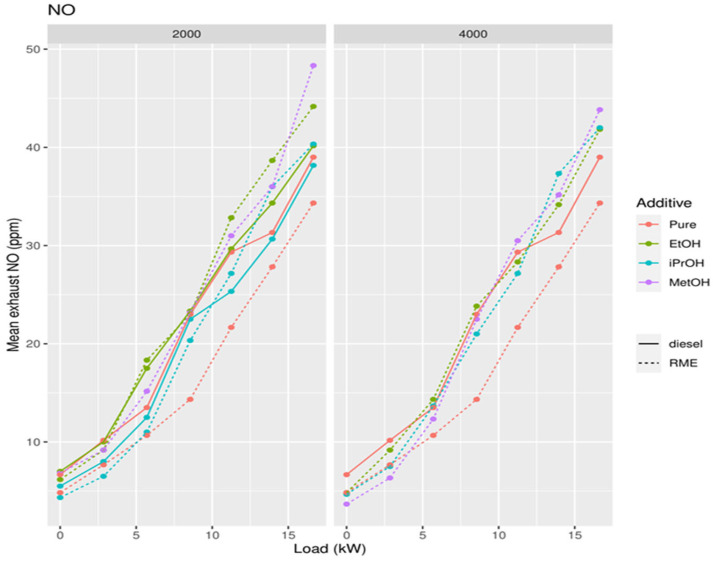
Concentration of NO in exhaust gases in function of the power of the diesel generator after addition of 2000 and 4000 ppm of methanol, ethanol, and isopropanol to biodiesel.

**Figure 12:**
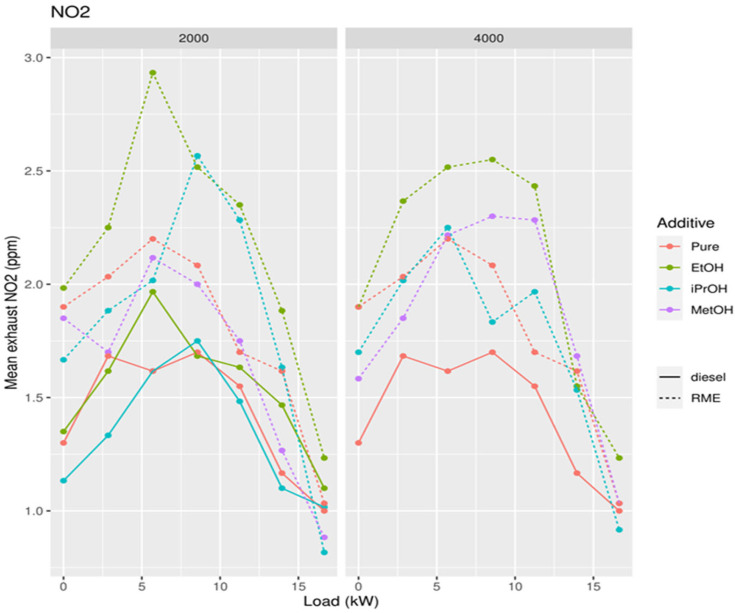
Concentration of NO_2_ in exhaust gases in function of the power of the diesel generator after addition of 2000 and 4000 ppm of methanol, ethanol, and isopropanol to biodiesel.

**Figure 13:**
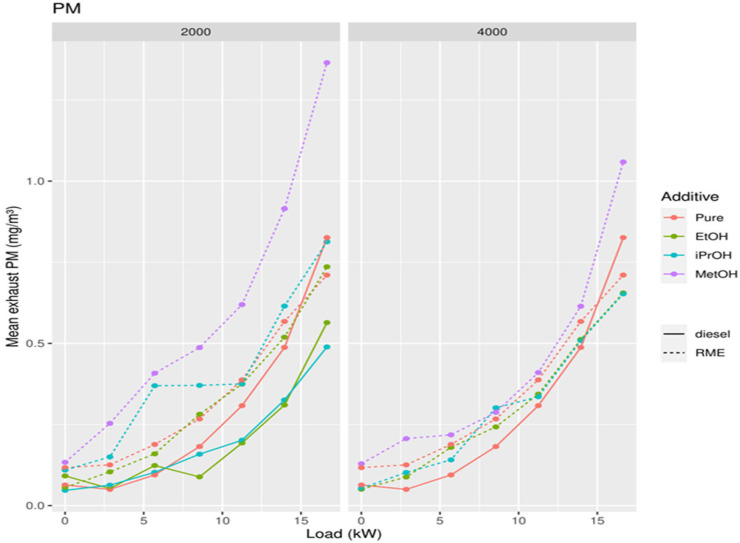
Concentration of PM in exhaust gases in function of the power of the diesel generator after addition of 2000 and 4000 ppm of methanol, ethanol, and isopropanol to biodiesel.

For biodiesel, similar techniques were used as before when alcohol was added to diesel. Exhaust values were recorded for NO, NO_2_, and PM for different mixtures of RME with alcohol additives (Figure 11, Figure 12 and Figure 13). Alcohol additives included ethanol, methanol, and isopropanol, at concentrations of 2000 ppm and 4000 ppm.

Exhaust measurements were carried out in duplicate at 7 different loads (0 kW (idle), 0.65 kW, 1.31 kW, 1.97 kW, 2.59 kW, 3.21 kW, and 3.83 kW) and for different mixtures of RME with alcohol additives. To obtain a summary value, describing the exhaust of a particular condition across the entire range of powers, the exhaust was plotted versus the load for each experiment and the area under the curve (AUC) was calculated. These AUC values for each condition served as input for the subsequent statistical analyses. A higher AUC corresponds to a higher exhaust value across the power levels tested. For each condition, six AUC values were available for statistical testing. The AUC values were compared between conditions using a one-way ANOVA test, followed by a posthoc analysis using Bonferroni’s correction for multiple hypothesis testing. The comparison between diesel and the RME mixtures, using diesel as standard, is summarized in Table S6 for NO, in Table S7 for NO_2_, and Table S8 for PM. The AUC value from the initial one-way ANOVA showed differences in AUC between the mixtures for NO, NO_2_ and PM.

**Figure 14:**
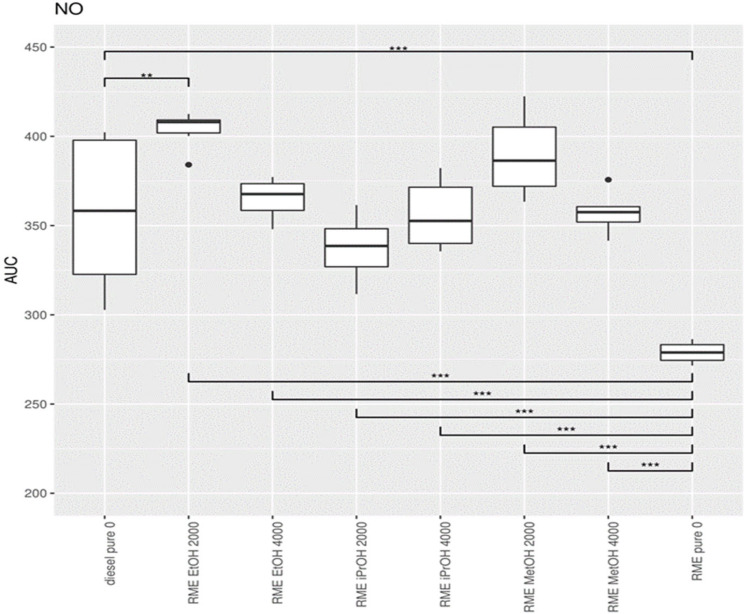
Boxplot of the statistical analysis of the concentration of NO in the exhaust gas. The center lines show the mean AUC value, obtained through bootstrap resampling. Asterisks show the significance of the pairwise comparison of the AUC values ** = *p* < 0.01; *** = *p* < 0.001).

**Figure 15:**
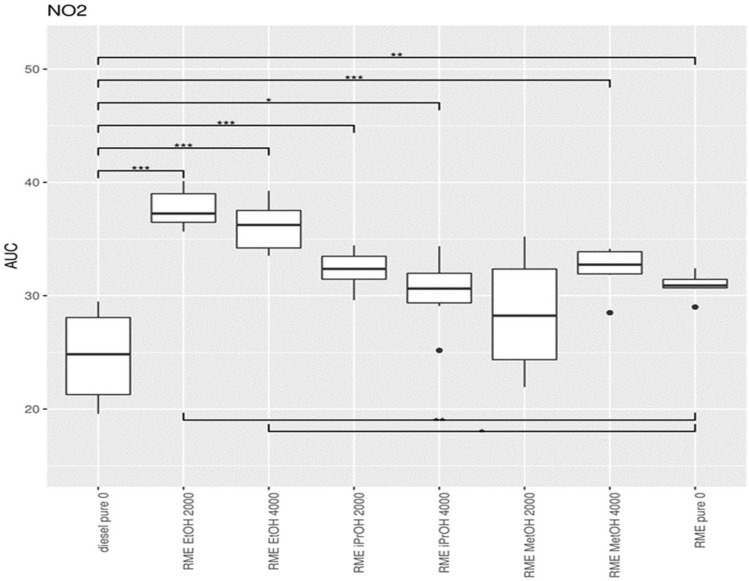
Boxplot of the statistical analysis of the concentration of NO_2_ in the exhaust gas. The center lines show the mean AUC value, obtained through bootstrap resampling. Asterisks show the significance of the pairwise comparison of the AUC values (* = *p* < 0.05; ** = *p* < 0.01; *** = *p* < 0.001).

**Figure 16:**
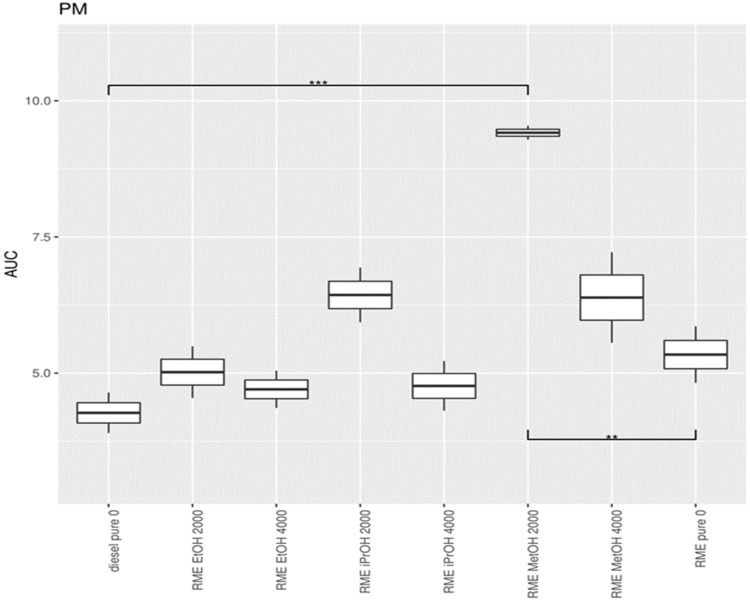
Boxplot of the statistical analysis of the concentration of PM in the exhaust gas. The center lines show the mean AUC value, obtained through bootstrap resampling. Asterisks show the significance of the pairwise comparison of the AUC values ** = *p* < 0.01; *** = *p* < 0.001).

The lowest NO concentration in the exhaust gas occurs when no alcohol is added (Figure 14). Pure RME shows the lowest NO concentration in the exhaust compared to pure diesel and all RME alcohol mixtures. Pure diesel shows the lowest concentration of NO_2_ in the emissions compared to all RME-alcohol mixtures, including RME. Pure RME showed a lower NO_2_ emission compared to the RME-alcohol mixtures, although the difference was not always significant (Figure 15). This suggests it would be better not to add alcohol to RME to lower concentrations of NO_2_. Considering the results of NO concentrations, it is clear that by adding an alcohol to RME NOx, emissions do not decrease, but increase. Pure diesel shows better results in terms of NO_2_ emissions than RME. For PM, a significantly large increase was obtained only after addition of 2000 ppm methanol, in comparison to both RME and diesel (Figure 16). 

## 4. Discussion

### 4.1. Adding Oxygenates to Mineral Diesel

To summarize, the lowest concentration of NO in the exhaust gas is found when 2000 ppm methanol is added. A concentration of 2000 ppm isopropanol as additive gives the lowest NO_2_ concentration in the exhaust gas, and if we consider NOx, in our case the sum of NO and NO_2_, we should use 2000 ppm of methanol to reach the lowest concentration in the exhaust gas. The lowest concentration of PM is reached when adding 2000 ppm ethanol to diesel. Ethanol performs best for PM, showing a decrease of 63% in PM (when using the mean AUC as a benchmark; Table 3). For NO_2_, methanol gives the best result with a decrease of NO_2_ 50%, and for NO, methanol gives the best result with a decrease of 56%. The question remains as to what additive can be used to decrease all the components at the same time.

This leads to a choice of additive that may be a mixture of different additives in order to lower, as much as possible, the concentrations of both PM and NOx. Literature concerning the use of oxygenates added to fuel illustrates that in [20], some functional groups have been tested on several different combustion characteristics, among others the exhaust gas composition, and more in particular the NOx and PM content.

NOx emission seemingly decreased with increasing carbon chain length of the oxygenate because the carbon chain length is suggested to decrease ignition delay by increasing length [20]. Additionally, total mass of PM increases and the number of particles decreases with carbon chain length. The article states that the ignition delay has a significant effect on combustion characteristics and emissions. The most important molecular features affecting ignition delay seemed to be threefold: the higher electronegativity of a fuel bound oxygen atom compared to a fuel carbon atom, the way an oxygen atom is bonded to the carbon chain, and the location of the oxygenated functional group in the molecule. These are important results. We have been studying oxygenates with the lowest carbon chain length possible, that are fluid under normal circumstances, and are thus able to make a solution in diesel. Next, the results of the measurements were compared only to the functionality of the oxygenated group. In this case, the alcohol function gave the best results.

In [21], the emissions of combustion of pure pentanol are compared to those of diesel and they seem to reach the same conclusion, namely that pentanol shows larger ignition delay and lower NOx and PM concentrations in the exhaust gases. The same authors find in [4] that adding pentanol to diesel/biodiesel blends lowers the NOx concentrations. The most important features affecting NOx concentrations are temperature and local air/fuel ratio. This is attributed firstly to the higher latent heat of vaporization which leads to lower combustion temperatures, thus reducing NOx, and secondly to the lower cetane number and thus longer ignition delay of pentanol, which will increase the concentration of NOx. In [22], the authors saw a decrease in PM when adding pentanol due to the higher ignition delay of pentanol and thus obtaining better premixing and a complete combustion.

Diesel/pentanol blends were also tested and the results were shown in [23]. The authors seem to get comparable results, borne out by the fact that increasing the content of pentanol seems to increase the NOx concentration. We also showed that, in our case, adding 2000 ppm alcohol showed better results than adding 4000 ppm. On the other hand, their measurements on PM shows that increasing the concentration of pentanol generates a reduction in soot formation.

In addition, in [24], the results of measuring emissions due to combustion in a diesel engine of blends of alcohol with diesel/biodiesel revealed a decrease in PM and NOx concentrations when adding ethanol or n-butanol.

If we look at [25], we see that branched molecules such as isopropanol tend to form more soot then unbranched, which is also confirmed in these experiments.

Using a filtering method, the results of the best oxygenate function seemed to be the alcohol function. In [30], NOx concentrations in the exhaust gas of ethanol-diesel blends were measured. In this case there was also a decrease in NOx, although higher concentrations of alcohol were used.

In [30], a reduction of harmful emissions of NOx and PM was also observed when adding 15% volume ethanol or methanol to diesel, where methanol gave better results for PM reduction and ethanol for NOx reduction. This could be explained by the lower flame temperature and the higher ignition delay.

In [31], the author comes to the conclusion that adding alcohol improves the smoke opacity in exhaust gas; the higher the alcohol content, the lower the opacity. A difference of opacity was measured when using methanol and ethanol, where the latter was less efficient. This could be explained by the % mass content of oxygen in the molecule.

Contrastingly, it should also be mentioned that the NOx concentration, which was measured when alcohol was added, seemed to have increased. The quantity added to the diesel is 5 and 10% volume, which is much higher than the concentrations added in these experiments. In the literature there seems to be some discussion on the effect of adding alcohol, but then the concentrations seem to differ, as do the type of engines used in the experiments, thus making a general conclusion difficult. When looking for grounds for the change in exhaust gas composition due to the molecular structure of alcohol, the literature [20,32] seems to agree that ignition delay, the cooling effect of evaporation, and the presence of oxygen in the molecule are important factors which lead to the changes in NOx and PM concentrations.

### 4.2. Effect of Alcohol Addition to Biodiesel and Diesel

According to [33], adding propanol, n-butanol, or n-pentanol improves the quality of the exhaust gases due to a significant decrease in NOx. In [34], measurements were made on a blend of biodiesel/alcohol where alcohol was not added as an additive. In our case the alcohol added was in low concentration and methanol, ethanol, and propanol were added, whilst butanol and pentanol were not investigated. It might be that due to the low concentrations, a decrease in NOx concentrations did not occur. This could be due to the fact that the latent heat needed for evaporation was not enough to lower temperatures during combustion. Another reason could be that the cetane number (CN), which also plays a major role in combustion, does not change much by adding low concentrations. In [35], the role of adding alcohol and the change in CN was established, revealing that B100 has the highest CN. In [36], ternary blends of n-butanol, diesel, and microalgae spirulina biodiesel were tested, and results show that at low biodiesel/diesel (B1, B2, B3) blends, NOx emission was higher than at high biodiesel/diesel (B20) blends. The Bosch Smoke Number (BSN) shows an overall reduction in the exhaust gas. In [37], the results show the same conclusion where microalgae biodiesel was blended with mineral diesel in a B20 configuration, and NOx as well as PM content decreased. In [38], Punnai biodiesel was used to make blends of 10 v% and 20 v% with butanol, and results showed a decrease in NOx as well as PM.

In [33], a comprehensive review was made of the effects of alcohol in diesel and biodiesel. Methanol added to biodiesel shows lower concentrations of PM and NOx than diesel. Ethanol added to biodiesel shows higher NOx than diesel but lower PM concentrations. Propanol added to biodiesel shows both lower NOx as well as reduced PM concentrations. Butanol added to biodiesel shows higher NOx emissions than diesel, but on the other hand, lower concentrations of PM are measured.

## 5. Conclusions

The main goal of this research is to decrease concentrations of NOx and PM in the exhaust gases when using diesel and biodiesel. Given the results, we should consider using ethanol as an additive in mineral diesel because of the high improvement in exhaust gas composition. Ethanol performed very well for PM. To lower NOx concentrations in the exhaust gases, methanol could be considered. Compared to pure biodiesel, if there is a decrease when adding alcohol to biodiesel, the decrease is not very large and statistically not significant and, in most cases, adding alcohol even increases PM and NOx in the exhaust gases. This is due to the fact that biodiesel itself is already an oxygenate because of the presence of oxygen atoms in the fatty acid methyl ester molecules. In the end, we can say that biodiesel without adding oxygenates as an alternative fuel shows interesting opportunities for maritime transport. We should nevertheless aim for biofuels with a low ecological footprint produced from either waste, yeast, or microalgae.

## Figures and Tables

**Table 1:** Engine (JavacNanomag NM 7500 B (KM 186FA)) specifications.

Injection system	Direct injection
Type	Single cylinder
Cooling	Air cooled
Aspiration	Naturally aspirated
Bore (mm)	86
Stroke (mm)	70
Compression ratio	19

**Table 2:** Numerical values of some important physical properties of the different oxygenates and fuels. NA: Not applicable. (Data obtained from refs. [26,27,28,29]).

	Boiling Point (°C)	Flash Point (°C)	Evaporation Heat * (kJ/mol at 25 °C) ** (kJ/kg at 25 °C)
water	100	NA	43.98 *
acetone	56	−20	30.99 *
acetaldehyde	20.8	−38	25.47 *
diethyl ether	35	−20	27.1 *
methanol	64.96	11	37.43 *
ethanol	78.37	12	42.32 *
propanol	97.1	15	47.45 *
isopropanol	82	12	45.39 *
diesel	180–340	74	265 **
RME	369	91–135	250 **

**Table 3:** Results of the statistical analysis of adding 2000 ppm of methanol, ethanol, and isopropanol. The value of the content of NO, NO_2_, and PM left in the exhaust gases as absolute AUC.

	NO (Mean AUC)	NO_2_ (Mean AUC)	PM (Mean AUC)
methanol	18.71	8.11	2.70
ethanol	20.89	8.92	2.35
propanol	24.61	8.93	3.77
isopropanol	26.47	8.91	2.90
pure diesel	43.03	18.17	6.39

## Data Availability

Data will be provided after embargo of 1 year.

## Supplementary Materials

The following supporting information can be downloaded at: https://www.mdpi.com/article/10.3390/ijerph19137637/s1, Table S1: Results of the statistical analysis of the addition of 2000 ppm of oxygenates; Table S2: Results of the statistical analysis of the addition of 4000 ppm of oxygenates; Table S3: Results of the statistical analysis of the addition of 2000 ppm of methanol and ether; Table S4: Results of the statistical analysis of the addition of 4000 ppm of methanol and ether; Table S5: Results of the statistical analysis of adding 2000 ppm of methanol. ethanol, propanol and isopropanol; Table S6: NO exhaust (AUC); Table S7: NO_2_ exhaust (AUC); Table S8: PM exhaust (AUC); Figure S1: Concentrations of NO in the exhaust gas in function of the load; Figure S2: Concentrations of NO_2_ in the exhaust gas in function of the load; Figure S3: Concentrations of PM in the exhaust gas in function of the load.
