# Supplementary material for: Influence of Adding Low Concentration of Oxygenates in Mineral Diesel Oil and Biodiesel on the Concentration of NO, NO2 and Particulate Matter in the Exhaust Gas of a One-Cylinder Diesel Generator
Source: Int J Environ Res Public Health. 2022 Jun 22;19(13):7637. doi: 10.3390/ijerph19137637 (PMC9265865; doi:10.3390/ijerph19137637)
Supplement: Supplementary file 1 [file ijerph-19-07637-s001.zip › ijerph-1759002-supplementary.pdf]

Supplementary data

Values DIESEL

Table S1: Results of the statistical analysis of the addition of 2000 ppm of oxygenates

NO							
additive	ppm	exhaust	meanAUC	sdAUC	uplim	lowlim	pDunnet
diesel	2000	NO	43.05	3.04	49.01	37.09	NA
methanol	2000	NO	23.17	1.27	25.66	20.67	8.00E-09
acetaldehyde	2000	NO	22.28	1.33	24.88	19.67	1.90E-09
acetone	2000	NO	30.90	2.65	36.10	25.70	1.30E-02
ether	2000	NO	31.04	3.93	38.74	23.34	7.81E-02
water	2000	NO	42.53	2.19	46.83	38.23	1.00E+00
NO_2							
additive	ppm	exhaust	meanAUC	sdAUC	uplim	lowlim	pDunnet
diesel	2000	NO2	18.18	0.73	19.61	16.76	NA
methanol	2000	NO2	9.61	0.19	9.99	9.23	0.00E+00
acetaldehyde	2000	NO2	8.48	0.46	9.37	7.58	0.00E+00
acetone	2000	NO2	16.47	0.83	18.09	14.84	5.98E-01
ether	2000	NO2	12.26	0.37	12.98	11.54	1.70E-12
water	2000	NO2	16.90	0.46	17.80	15.99	6.73E-01
PM							
additive	ppm	exhaust	meanAUC	sdAUC	uplim	lowlim	pDunnet
diesel	2000	PM	6.42	1.49	9.34	3.51	NA
methanol	2000	PM	1.78	0.11	2.00	1.55	9.28E-03
acetaldehyde	2000	PM	2.87	0.23	3.31	2.43	9.17E-02
acetone	2000	PM	2.48	0.20	2.87	2.09	4.35E-02
ether	2000	PM	2.89	0.50	3.87	1.91	1.22E-01
water	2000	PM	3.83	0.27	4.36	3.29	4.30E-01

Table S2: Results of the statistical analysis of the addition of 4000 ppm of oxygenates

NO							
additive	ppm	exhaust	meanAUC	sdAUC	uplim	lowlim	pDunnet
diesel	4000	NO	43.18938	3.003659	49.07655	37.30221	NA
methanol	4000	NO	8.941252	2.227269	13.3067	4.575806	0
acetaldehyde	4000	NO	21.39546	1.873859	25.06822	17.7227	3.73E-09
acetone	4000	NO	20.3021	2.169698	24.55471	16.04949	3.27E-09
ether	4000	NO	20.59025	2.466486	25.42456	15.75593	3.04E-08
water	4000	NO	28.65808	3.129765	34.79242	22.52374	0.004043
NO_2							
additive	ppm	exhaust	meanAUC	sdAUC	uplim	lowlim	pDunnet
diesel	4000	NO2	18.17528	0.712494	19.57177	16.77879	NA
methanol	4000	NO2	5.723244	0.375965	6.460136	4.986351	0
acetaldehyde	4000	NO2	6.42347	0.191701	6.799203	6.047736	0
acetone	4000	NO2	11.06401	0.67471	12.38644	9.741576	2.13E-12
ether	4000	NO2	10.41871	0.361499	11.12725	9.710173	0
water	4000	NO2	14.14762	0.519174	15.1652	13.13004	2.45E-05
PM							
additive	ppm	exhaust	meanAUC	sdAUC	uplim	lowlim	pDunnet
diesel	4000	PM	6.382763	1.468186	9.260408	3.505119	NA
methanol	4000	PM	2.282876	0.455682	3.176012	1.389739	0.038268
acetaldehyde	4000	PM	2.630642	0.234553	3.090366	2.170919	0.058077
acetone	4000	PM	2.444337	0.24411	2.922792	1.965881	0.040703
ether	4000	PM	2.024345	0.523508	3.05042	0.99827	0.025859
water	4000	PM	3.044619	0.243632	3.522139	2.5671	0.124491

Table S3: Results of the statistical analysis of the addition of 2000 ppm of methanol and ether

NO							
additive	ppm	exhaust	meanAUC	sdAUC	uplim	lowlim	pDunnet
diesel	2000	NO	43.09	3.06	49.09	37.09	NA
methanol	2000	NO	18.77	2.27	23.21	14.32	3.40E-10
ether	2000	NO	18.74	1.48	21.65	15.84	1.63E-12
NO_2							
additive	ppm	exhaust	meanAUC	sdAUC	uplim	lowlim	pDunnet
diesel	2000	NO2	18.15	0.72	19.56	16.75	NA
methanol	2000	NO2	8.11	0.20	8.50	7.71	0.00E+00
ether	2000	NO2	8.96	0.38	9.72	8.21	0.00E+00
PM							
additive	ppm	exhaust	meanAUC	sdAUC	uplim	lowlim	pDunnet
diesel	2000	PM	6.36	1.45	9.20	3.53	NA
methanol	2000	PM	2.70	0.21	3.11	2.30	2.43E-02
ether	2000	PM	3.72	0.26	4.23	3.21	1.44E-01

Table S4: Results of the statistical analysis of the addition of 4000 ppm of methanol and ether

NO							
additive	ppm	exhaust	meanAUC	sdAUC	uplim	lowlim	pDunnet
diesel	4000	NO	43.00	3.09	49.05	36.95	NA
methanol	4000	NO	20.55	2.76	25.96	15.14	1.18E-07
ether	4000	NO	21.97	2.59	27.05	16.90	3.60E-07
NO_2							
additive	ppm	exhaust	meanAUC	sdAUC	uplim	lowlim	pDunnet
diesel	4000	NO2	18.14	0.75	19.61	16.67	NA
methanol	4000	NO2	8.42	0.27	8.95	7.90	0.00E+00
ether	4000	NO2	8.73	0.23	9.18	8.27	0.00E+00
PM							
additive	ppm	exhaust	meanAUC	sdAUC	uplim	lowlim	pDunnet
diesel	4000	PM	6.32	1.50	9.26	3.38	NA
methanol	4000	PM	3.88	0.17	4.22	3.55	2.12E-01
ether	4000	PM	3.60	0.15	3.89	3.31	1.41E-01

Table S5: Results of the statistical analysis of adding 2000ppm of methanol. ethanol.propanol and isopropanol

NO							
additive	ppm	exhaust	meanAUC	sdAUC	uplim	lowlim	pDunnet
diesel	2000	NO	43.02862	3.047513	49.00174	37.05549	NA
ethanol	2000	NO	20.88593	0.682071	22.22279	19.54907	5.35E-12
isopropanol	2000	NO	26.47124	2.978588	32.30927	20.6332	0.000409
methanol	2000	NO	18.71185	2.22365	23.07021	14.3535	4.60E-10
propanol	2000	NO	24.61347	0.576333	25.74309	23.48386	1.16E-08
NO_2							
additive	ppm	exhaust	meanAUC	sdAUC	uplim	lowlim	pDunnet
diesel	2000	NO2	18.17137	0.736289	19.61449	16.72824	NA
ethanol	2000	NO2	8.919609	0.170634	9.254051	8.585167	0
isopropanol	2000	NO2	8.908846	0.366406	9.627001	8.190691	0
methanol	2000	NO2	8.111031	0.203384	8.509665	7.712398	0
propanol	2000	NO2	8.933295	0.414293	9.74531	8.12128	0
PM							
additive	ppm	exhaust	meanAUC	sdAUC	uplim	lowlim	pDunnet
diesel	2000	PM	6.388562	1.468749	9.26731	3.509813	NA
ethanol	2000	PM	2.350732	0.181275	2.706031	1.995432	0.025452
isopropanol	2000	PM	2.895145	0.178778	3.24555	2.54474	0.072889
methanol	2000	PM	2.699136	0.203855	3.098693	2.299579	0.051371
propanol	2000	PM	3.765731	0.356122	4.463731	3.067731	0.330629

Values BIODIESEL

Table S6: NO exhaust (AUC)

*P-values were obtained via a posthoc analysis with Bonferroni correction for multiple hypothesis testing
** A positive value means that, summarized by the AUC across the measured power levels, mean exhaust from blend 1 is higher than blend 2.

p-value one-way ANOVA : 1.68E-10					
Posthoc analysis with Bonferroni correction					
			95% confidence interval		
	AUCdifference	SE	lowlim	uplim	pBonferroni
RME EtOH 2000 - diesel pure 0	46.44	12.50	21.94	70.94	7.17E-03
RME EtOH 4000 - diesel pure 0	8.00	12.50	-16.50	32.50	1.82E+00
RME iPrOH 2000 - diesel pure 0	-19.75	12.50	-44.25	4.75	8.57E-01
RME iPrOH 4000 - diesel pure 0	-1.13	12.50	-25.63	23.38	1.86E+00
RME MetOH 2000 - diesel pure 0	32.30	12.50	7.80	56.80	1.30E-01
RME MetOH 4000 - diesel pure 0	0.11	12.50	-24.39	24.61	1.86E+00
RME pure 0 - diesel pure 0	-78.31	12.50	-102.81	-53.81	9.27E-07
RME EtOH 2000 - RME pure 0	124.75	12.50	100.25	149.25	4.67E-13
RME EtOH 4000 - RME pure 0	86.31	12.50	61.81	110.81	5.40E-07
RME iPrOH 2000 - RME pure 0	58.56	12.50	34.06	83.06	4.18E-04
RME iPrOH 4000 - RME pure 0	77.19	12.50	52.69	101.69	3.17E-06
RME MetOH 2000 - RME pure 0	110.61	12.50	86.11	135.11	4.43E-11
RME MetOH 4000 - RME pure 0	78.43	12.50	53.92	102.93	3.30E-06

Table S7: NO_2 exhaust (AUC)

p-value one-way ANOVA : 9.75E-8					
Posthoc analysis with Bonferroni correction					
			95% confidence interval		
	AUCdifference	SE	lowlim	uplim	pBonferroni
RME EtOH 2000 - diesel pure 0	13.02	1.74	9.60	16.43	8.32E-09
RME EtOH 4000 - diesel pure 0	11.46	1.74	8.04	14.88	3.71E-07
RME iPrOH 2000 - diesel pure 0	7.63	1.74	4.21	11.05	8.58E-04
RME iPrOH 4000 - diesel pure 0	5.70	1.74	2.28	9.11	2.10E-02
RME MetOH 2000 - diesel pure 0	3.74	1.74	0.32	7.16	2.87E-01
RME MetOH 4000 - diesel pure 0	7.67	1.74	4.26	11.09	7.35E-04
RME pure 0 - diesel pure 0	6.24	1.74	2.83	9.66	9.25E-03
RME EtOH 2000 - RME pure 0	6.77	1.74	3.36	10.19	4.28E-03
RME EtOH 4000 - RME pure 0	5.22	1.74	1.80	8.63	4.94E-02
RME iPrOH 2000 - RME pure 0	1.39	1.74	-2.03	4.80	1.00E+00
RME iPrOH 4000 - RME pure 0	-0.55	1.74	-3.96	2.87	1.00E+00
RME MetOH 2000 - RME pure 0	-2.50	1.74	-5.92	0.91	1.00E+00
RME MetOH 4000 - RME pure 0	1.43	1.74	-1.99	4.85	1.00E+00

Table S8: PM exhaust (AUC)

**p-value one-way ANOVA : 0.0013**					
**Posthoc analysis with Dunnett correction**					
			95% confidence interval		
	AUCdifference	SE	lowlim	uplim	pBonferroni
RME EtOH 2000 - diesel pure 0	0.75	0.69	-0.61	2.11	1.31E+00
RME EtOH 4000 - diesel pure 0	0.43	0.69	-0.93	1.79	1.00E+00
RME iPrOH 2000 - diesel pure 0	2.16	0.69	0.81	3.52	1.03E-01
RME iPrOH 4000 - diesel pure 0	0.49	0.69	-0.86	1.85	1.00E+00
RME MetOH 2000 - diesel pure 0	5.14	0.69	3.79	6.50	6.79E-04
RME MetOH 4000 - diesel pure 0	2.12	0.69	0.76	3.47	1.13E-01
RME pure 0 - diesel pure 0	1.07	0.69	-0.29	2.43	8.51E-01
RME EtOH 2000 - RME pure 0	-0.32	0.69	-1.68	1.04	1.00E+00
RME EtOH 4000 - RME pure 0	-0.64	0.69	-2.00	0.72	1.00E+00
RME iPrOH 2000 - RME pure 0	1.10	0.69	-0.26	2.45	9.34E-01
RME iPrOH 4000 - RME pure 0	-0.58	0.69	-1.93	0.78	1.00E+00
RME MetOH 2000 - RME pure 0	4.07	0.69	2.72	5.43	3.70E-03
RME MetOH 4000 - RME pure 0	1.05	0.69	-0.31	2.40	1.00E+00

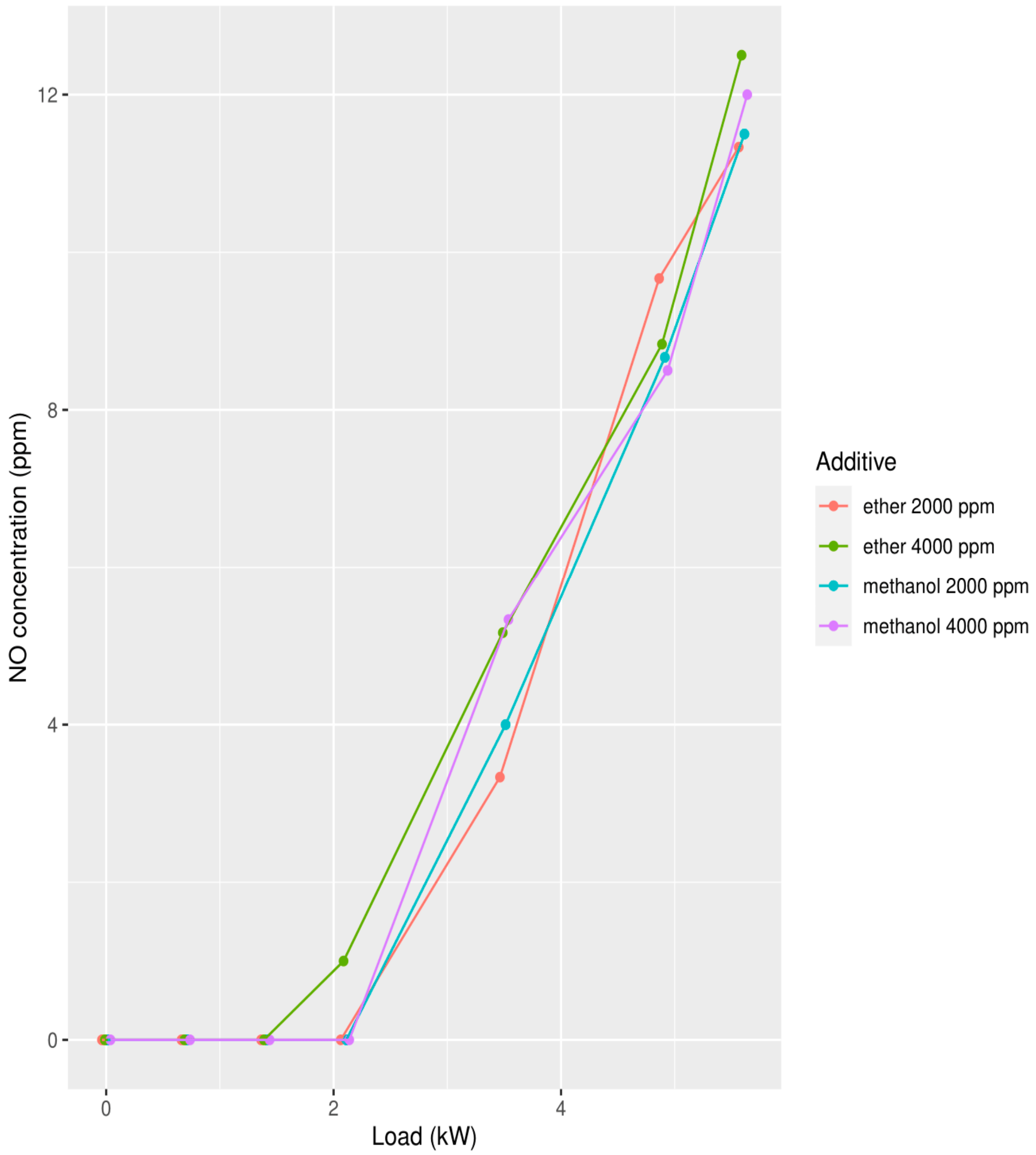

Figure S1 : Concentrations of NO in the exhaust gas in function of the load

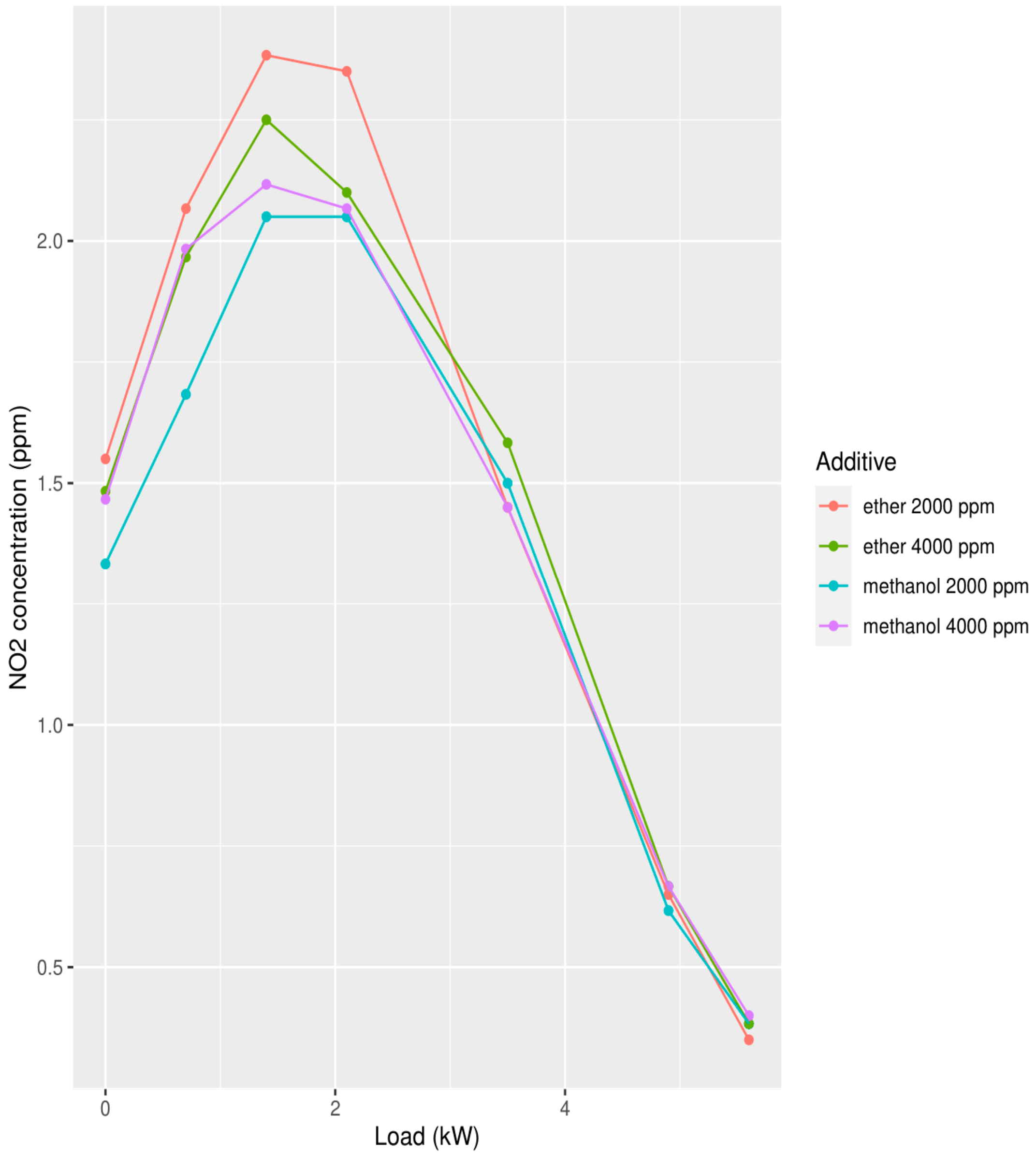

Figure S2: Concentrations of NO_2 in the exhaust gas in function of the load

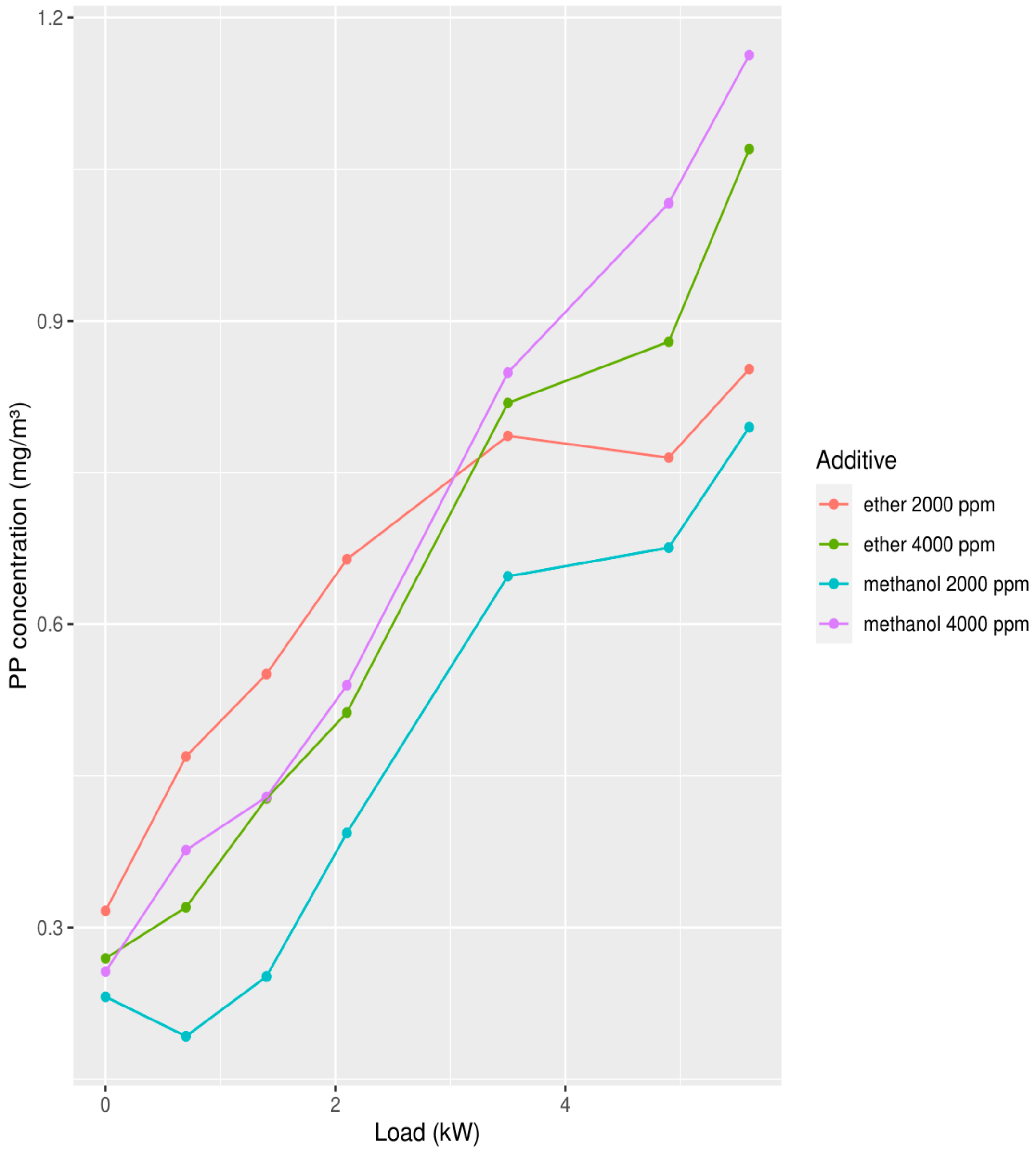

Figure S3: Concentrations of PM in the exhaust gas in function of the load
